# Retrograde Intrarenal Renal Surgery versus Supine Mini Percutaneous Nephrolithotripsy in Treatment of Renal Pelvic Stones Less than 2 cm, Randomized Clinical Study

**DOI:** 10.5152/tud.2026.25121

**Published:** 2026-03-13

**Authors:** Ahmed Hakim Abdelgawad, Mamdouh Abdelhamid Elhawy, Al Ayman Hussein Fathy Hussein, Tarek KH. Fathelbab, Amr Kamal Rabea Tolba, Ahmed Mohamed Fawzy

**Affiliations:** Department of Urology, Minia University Faculty of Medicine, Egypt

**Keywords:** Mini percutaneous nephrolithotomy, renal pelvis stones, retrograde intrarenal renal surgery, treatment

## Abstract

**Objective:**

Retrograde intrarenal surgery (RIRS) is the first-line treatment for kidney stones between 1 and 2 cm and can serve as an alternative to percutaneous nephrolithotomy for larger stones in high-risk patients, including those with bleeding disorders, obesity, renal congenital abnormalities, or solitary kidneys. We aimed to compare the safety and efficacy of supine mini-percutaneous nephrolithotomy (PCNL) versus RIRS for the treatment of renal pelvic stones less than 2 cm.

**Methods:**

This prospective, randomized, double-blinded study included 50 patients aged >18 years, of both sexes, with renal stones <2 cm. Patients were randomized into 2 equal groups: Group A underwent supine mini-PCNL, while Group B underwent RIRS. Stone and patient characteristics, SFR, and perioperative events were compared between groups.

**Results:**

A total of 50 patients equally distributed into the 2 groups. Operative time was significantly lower in Group A than in Group B (*P* < .05), while fluoroscopic time was significantly higher in Group A than in Group B (*P* < .05). Also, hospital stay was significantly longer in Group A compared to Group B (*P* < .001). SFR and retreatments were comparable between both groups. Complications were also similar, with no significant differences observed.

**Conclusions:**

Supine mini-PCNL remains an effective option for achieving high stone clearance with a shorter operative time while avoiding ureteral access manipulation, while RIRS provides advantages such as reduced radiation exposure, shorter hospitalization but with a higher need for stenting and secondary procedures. Supine mini-PCNL is not inferior to RIRS in the treatment of less than 2 cm renal stones.

**Main Points**

- Supine mini-percutaneous nephrolithotomy (PCNL) achieved comparable stone-free rates (92%) to retrograde intrarenal surgery (RIRS) (84%) for renal pelvic stones <2 cm.
- Mini-PCNL demonstrated significantly shorter operative time but required a longer hospital stay and higher fluoroscopic exposure.
- Retrograde intrarenal surgery (RIRS) offered advantages of lower radiation exposure and shorter hospitalization, but all patients required DJ stent placement.
- Mini-PCNL bypassed ureteral access, reducing the risk of ureteral injury and lowering intrapelvic pressure.
- Both procedures were safe with similar complication rates, supporting mini-PCNL as a viable alternative to RIRS for small renal stones.

## Introduction

Retrograde intrarenal surgery (RIRS) was first reported for the treatment of small kidney stones in 2002. In recent years, urologists have also suggested using this approach for larger stones due to its lower complication rates and reduced morbidity.^1^

RIRS offers several advantages, including minimal invasiveness, safety, rapid recovery, and high efficiency. As it is performed through a natural orifice under direct vision, it is particularly effective for kidney stones ≤ 2 cm in diameter, as well as complex kidney stones. Additionally, RIRS is suitable for elderly patients, obese individuals, patients with hemorrhagic disorders, and those who are not candidates for extracorporeal shock wave lithotripsy (ESWL) or percutaneous nephrolithotomy (PCNL).^2^ Given its ability to minimize significant morbidities associated with percutaneous approaches, RIRS has gained widespread attention.^3^ The European Association of Urology (EAU) guidelines recommend RIRS as the standard treatment option for small-to-medium-sized (≤ 2 cm) renal stones.^4^^,^^5^ However, RIRS still carries a risk of infection with elevation of the intrarenal pelvic pressure, repeated procedures especially in tight ureters, higher risk of equipment failure including ureteroscope damage, stuck ureteroscope or ureteral access sheath entrapment, and need for multiple procedures.^6^

Percutaneous nephrolithotomy (PCNL) is currently the preferred first-line therapy for large radiopaque and large cystine renal stones that are not amenable to ESWL.^7^ Recently, the use of miniaturized instruments in PCNL has gained popularity due to its potential to reduce treatment-related complications while maintaining good clinical outcomes. Mini-PCNL has been shown to be as effective as standard PCNL in terms of stone clearance,^8^ while also being associated with lower blood loss and reduced transfusion rates.^9^ However, limitations of standard PCNL include longer operative time, decreased visibility,^10^ and challenges in stone retrieval.^11^

To address these limitations, new technologies have been introduced to enable simultaneous irrigation flow under low-pressure conditions. One such advancement is the minimally invasive PCNL (MIP) system (Karl Storz & Co. KG, Tuttlingen, Germany)®, which was designed to operate as an open low-pressure irrigation system and facilitates stone clearance through the vacuum cleaner effect.^12^ Several studies have confirmed the efficacy of MIP, demonstrating comparable stone-free (SF) rates with standard PCNL while achieving lower rates of major complications.^13^^-^^16^

The study aimed to compare the safety of mini-PNL over RIRs in bypassing ureteric access and low-pressure irrigation in supine mini-PNL and efficacy, particularly the stone-free rate of the Supine Mini-PCNL, which benefits from gravity-assisted clearance and the vacuum cleaner effect, with RIRS for the treatment of renal pelvic stones ≤ 2 cm.

## Material and Methods

This prospective, randomized, double-blinded study was conducted on 50 patients aged over 18 years, of both sexes, presenting with unilateral surgery-naïve renal stones measuring ≤2 cm. Patients with any distal obstruction or associated congenital anomalies were excluded from the study. Patients with concurrent infection were also postponed until treatment or excluded due to refusal. The study was conducted from September 2022 to November 2024 after obtaining approval from the local institutional ethical committee. Informed written consent was obtained from all participants before their enrollment.

### Randomization and Blinding

An online randomization program (http://www.randomizer.org) was used to generate a randomized list. Each patient was assigned a unique code by the hospital statistical unit, which was placed in an opaque sealed envelope to ensure allocation concealment. Patients were randomly allocated in a 1:1 ratio into 2 parallel groups: Group A, which underwent Mini-PCNL, and Group B, which underwent RIRS. Blinding of patients and surgeons was not feasible. However, to minimize potential bias, data collectors as well as data analysts were blinded to group allocation. Postoperative data, including stone-free rates, complications, and hospital stay, were collected and analyzed by individuals who were not involved in the surgical procedures and were unaware of the treatment assignments.

### Preoperative Evaluation

All patients underwent a comprehensive preoperative assessment, including detailed history-taking and physical examination. Laboratory investigations were performed as per standard protocols. Radiological evaluations included pelvic-abdominal ultrasound, plain kidney-ureter-bladder (KUB) X-ray, and a computed tomography (CT) scan of the KUB region. These imaging modalities were used to assess the stone burden, anatomical variations, and any underlying pathology that could influence the surgical approach.

### Group I: Mini-PCNL Procedure

#### Anesthesia and Patient Positioning:

A single dose of a broad-spectrum prophylactic antibiotic, specifically a third-generation cephalosporin, was administered intravenously 1 hour before surgery. All patients underwent general anesthesia with endotracheal intubation. After anesthesia induction, a ureteric catheter was inserted under fluoroscopic guidance, and the patient was placed in a modified supine position with a cushion placed under the abdomen to achieve a flank-free position. Standard sterilization and draping techniques were followed before proceeding with the procedure.

#### Percutaneous Access and Tract Dilation:

The collecting system was opacified by injecting contrast material through the ureteric catheter. Under fluoroscopic guidance in the vertical (anteroposterior) position, an 18-gauge Chiba needle was percutaneously inserted to access the desired calyx. To confirm the appropriate depth, the C-arm was tilted 30° toward the patient’s head, ensuring that the needle was neither too superficial nor too deep. Correct placement within the pelvicalyceal system was verified by observing respiratory movement of the needle and the appearance of urine backflow. A 0.035 Fr guidewire was introduced through the needle and manipulated within the collecting system to facilitate subsequent tract dilation. The lumbo-dorsal fascia was then punctured and split using a long hemostat under fluoroscopic guidance.

A one-step metallic dilator was advanced over the guidewire, followed by the insertion of a 15/18 Fr metallic sheath. A 12 Fr semi-rigid nephoscope (Storz Miniperc System) was introduced into the collecting system to visualize the anatomy and localize the stone.

#### Stone Fragmentation and Removal:

Once the stone was identified, fragmentation was performed using a high-power Ho:YAG laser (Sphinx 100 W) with a 365-µm fiber using laser variables at 1-1.5 J and 10-15 Hz frequency. The dusting technique was employed to break the stone into fine particles, which were subsequently removed using the vacuum cleaner effect. Upon completion of the procedure, a ureteral catheter was either retained or a double-J (DJ) stent was inserted based on intraoperative findings.

#### Postoperative Care:

A 12 Fr Nelaton catheter was inserted as a nephrostomy tube, and its position was confirmed fluoroscopically. The ureteral catheter was kept in place for 3-5 days, secured to the urethral catheter, while the nephrostomy tube was removed once urine became clear, typically within 24-48 hours. A plain KUB X-ray was obtained, usually on the second postoperative day, to confirm stone clearance. The DJ stent was maintained for 2-4 weeks postoperatively.

### Group II: Retrograde Intrarenal Surgery Procedure

#### Anesthesia and Patient Positioning:

Prophylactic antibiotics and general anesthesia were administered in the same manner as in the Mini-PCNL group. The procedure was performed with the patient in the dorsal lithotomy position. The bladder was accessed using either a cystoscope or a semi-rigid ureterorenoscope.

#### Ureteral Dilatation and Access Sheath Placement:

A guidewire was passed into the renal pelvis under fluoroscopic guidance once the ureteral orifice was visualized. Ureteral dilatation was performed using Teflon dilators in sequential increments from 6 Fr up to 14 Fr. Following adequate dilatation, a ureteral access sheath (UAS) of 11/13 Fr was introduced under fluoroscopic guidance. In 3 cases, the UAS could not reach the renal pelvis due to a tight ureter, requiring the placement of a DJ stent for passive ureteral dilatation.

#### Stone Fragmentation and Removal:

Once access was achieved, a flexible ureteroscope (9.5 Fr LithoVue, Boston Scientific) was advanced through the UAS to reach the renal pelvis. Stone fragmentation was performed using a high-power Ho:YAG laser (Sphinx 100 W) with a 272-µm fiber at 0.6-1.2 J adjusted to a frequency of 10-20 Hz. The dusting technique was used to break down the stone into fine particles, which were subsequently removed using a Dormia basket. A DJ stent was placed in all cases and was removed 3-4 weeks postoperatively.

#### Postoperative Care:

The operative duration was recorded from the time of cystoscopy initiation to DJ stent fixation. Postoperative evaluation included a KUB X-ray and abdominal ultrasound on the first postoperative day and at 2 weeks to assess stone clearance as the primary outcome and identify any potential complications and retreatment as secondary outcomes. Patients with residual stones > 4 mm or clinically relevant postoperative events had CT KUB for confirmation and management.

### Study Power and Statistical Analysis

Sample size was not calculated because the study protocol was to take all eligible cases in a specific predefined period, so it was not feasible to calculate a priori sample size. Therefore, we calculated the study power while assuming a moderate Cohen’s d effect size of 0.4 to be detected between study groups, with a level of significance proposed at 0.05 and a total of 50 patients. The power of the study was estimated to be 80%, so this study can be described as exploratory. Post hoc analysis is only descriptive for future studies. Statistical analysis was performed using SPSS, version 27 (IBM©, Armonk, NY, USA). The normality of data distribution was assessed using the Shapiro–Wilk test and histogram analysis. Quantitative parametric data were expressed as mean ± standard deviation (SD) and analyzed using an unpaired Student’s *t*-test. Quantitative non-parametric data were presented as median and interquartile range (IQR) and analyzed using the Mann–Whitney *U*-test. Qualitative variables were reported as frequencies and percentages and analyzed using the chi-square test or Fisher’s exact test, as appropriate. A 2-tailed *P*-value <.05 was considered statistically significant.

### Ethical Consideration

Patients were provided with video-illustrated and well-informed written consent, as required by the institutional review board. The study was approved by the Institutional Review Board, IRB No. 00008718, Decision No. 111/2022.

## Results

**Figure 1.**
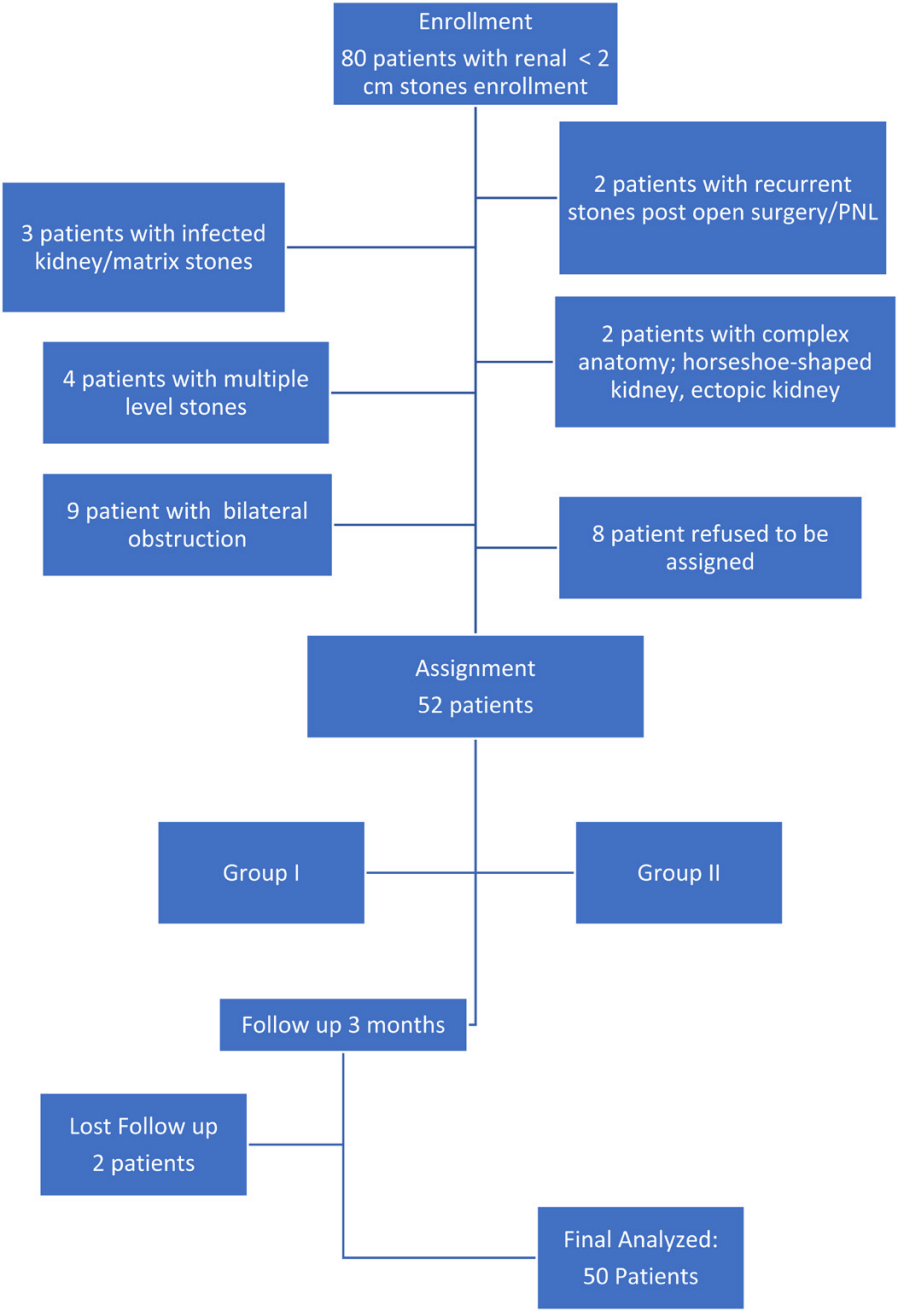
Flowchart of the studied group.

This study was conducted on 80 patients diagnosed with renal stones and scheduled for surgical intervention at the outpatient clinic of the Urology Department, Urology & Nephrology University Hospital. Out of those patients, 52 registered participants with 2 patients lost follow up (figure 1). The patients were randomized into 2 groups: 25 patients underwent supine Mini-PCNL (Group I), while another 25 patients underwent RIRS (Group II). Two patients were lost to follow-up and were subsequently excluded from the final analysis (Figure 1).

### Comparison of Baseline Characteristics and Operative Data

The baseline demographic data and stone characteristics were comparable between both study groups. There were no statistically significant differences in age, sex distribution, laterality (right vs. left kidney involvement), stone size, stone density (HU), or body mass index (BMI), as shown in Table 1. However, the operative time was significantly lower in Group A (Mini-PCNL) compared to Group B (RIRS) (80.6 ± 11.8 min vs. 93.6 ± 11.6 min, *P* < .001). Conversely, fluoroscopic time was significantly higher in Group A than in Group B (334.6 ± 55.7 sec vs. 68.8 ± 31.5 sec, *P* < .001). This indicates that while Mini-PCNL was a quicker procedure overall, it required more fluoroscopic guidance than RIRS.

### Postoperative Outcomes

The mean hemoglobin drop postoperatively was slightly higher in Group B (RIRS) at 0.68 ± 0.27 g/dL compared to 0.58 ± 0.18 g/dL in Group A (Mini-PCNL), although this difference was not statistically significant (*P* = .12). However, Group A had a significantly longer hospital stay (3.2 ± 0.87 days) compared to Group B (1.52 ± 0.58 days), with a *P*-value of < .001. Regarding postoperative stent placement, all patients in Group B (100%) received a double-J (DJ) stent, whereas in Group I, only 16% received DJ stents, and the majority (84%) had ureteral stents. This difference in stent usage was statistically significant (*P* < .001). These findings suggest that while both procedures had comparable effects on hemoglobin levels, RIRS was associated with a shorter hospital stay and faster postoperative recovery (Table 2).

### Stone-Free Rate and Need for Auxiliary Treatment

The stone-free rate, defined as no residual stone fragments or fragments smaller than 4 mm in diameter, was high in both groups. In Group I, 22 patients (92%) achieved complete stone clearance, while 2 patients (8%) had significant residual stones requiring extracorporeal shock wave lithotripsy (SWL) as auxiliary treatment. In Group II, 21 patients (84%) were stone-free, while 4 patients (16%) required SWL for residual stones. There was no statistically significant difference between the 2 groups regarding stone-free rates and the need for auxiliary treatment (*P* > .05) (Table 3).

### Postoperative Complications

Complication rates, assessed using the Modified Clavien Grading System, were comparable between the 2 groups (Table 4). The most common postoperative complications included fever (16% in Group A vs. 12% in Group II, *P* = .72) and transient hematuria. Blood transfusion was not needed in either group. Urinary tract infection (UTI) was noted in 1 patient (4%) in Group II, but none in Group A (*P* = .23). Additionally, urine leakage lasting more than 12 hours after removal of urinary catheters occurred in 2 patients in Group I, which was resolved spontaneously while keeping ureteric catheters.

In terms of complication severity, Grade A complications occurred in 6 patients (24%) in Group A and in 3 patients (12%) in Group II, with no statistically significant difference (*P* = .39). These findings indicate that both Mini-PCNL and RIRS are safe and effective procedures, with a similar overall complication profile.

## Discussion

Renal stones are among the most common urological conditions encountered in clinical practice, with a rising incidence globally. The optimal treatment approach depends on various factors, including stone size, location, composition, patient anatomy, and the presence of any associated symptoms or complications.^1^ Minimally invasive surgical options have revolutionized stone management, offering effective stone clearance with reduced morbidity. Two widely adopted modalities for treating renal pelvic stones are RIRS and Mini-PCNL.^2^

RIRS, a retrograde approach utilizing flexible ureteroscopes, is known for its minimal invasiveness, shorter recovery time, and suitability for anatomically complex kidneys.^4^ On the other hand, Mini-PCNL, a modified version of the conventional PCNL with reduced tract size, offers high stone clearance rates while minimizing bleeding and parenchymal injury.^7^ Despite their increasing use, there remains an ongoing debate about the comparative efficacy, safety, and postoperative outcomes of these techniques, especially for stones less than 2 cm in size.^9^^-^^11^ Therefore, this study aimed to evaluate and compare Mini-PCNL and RIRS in terms of operative time, fluoroscopy exposure, hospital stay, complications, and stone-free rates in a randomized clinical setting.

The findings revealed that the mean operative time (OT) in Group A was 80.6 ± 11.8 minutes, which was significantly shorter than in Group B (93.6 ± 11.6 minutes). Starting from the cystoscopy until the completion of the procedure, this difference may have resulted from frequent withdrawal of the nephroscope, suction for clearance of residual dust, to enhance vision, and to lower the intrarenal pressure. This resulted in a higher OT in Group B with a highly statistically significant difference. In agreement with the results, Dhayal et al^17^ reported a significantly lower mean operative time in the Mini-PCNL group (32.3 ± 12.65 minutes) compared to the RIRS group (51.2 ± 8.63 minutes). Similarly, Kanchi et al^18^ found that the mean operative time in the Mini-PCNL group was 44.07 ± 9.05 minutes, whereas in the RIRS group, it was 72.23 ± 11.01 minutes, showing a statistically significant difference. However, contrasting results were reported by Mahmood et al,^19^ who observed that the mean operative time in the Mini-PCNL group (97 ± 37 minutes) was slightly higher than that in the RIRS group (86 ± 82 minutes), but without a statistically significant difference between the 2 procedures.^20^ Also, recent studies reported lower OT in RIRs groups^20^^-^^22^ which may be due to different operative time definitions and different lithotripsy techniques.

Regarding fluoroscopic time, the study showed that the mean fluoroscopic time in Group A was significantly higher (334.6 ± 55.7 seconds) than in Group B (68.8 ± 31.5 seconds). This is consistent with findings by Sebaey et al,^23^ who reported that the mean fluoroscopic time in the Mini-PCNL group (486.6 ± 123 seconds) was significantly higher than in the RIRS group (348 ± 118.8 seconds). Likewise, Shazly et al^24^ and Kaya et al^25^ found that the fluoroscopic time was significantly longer in Mini-PCNL compared to RIRS. A recent prospective randomized trial reported similar results (273 ± 166 seconds in miniperc group vs. 49 ± 38 seconds in RIRS group).^20^

In terms of postoperative outcomes, the mean hemoglobin drop in Group A was 0.58 ± 0.18 g/dL, slightly lower than in Group B (0.68 ± 0.27 g/dL), with no statistically significant difference. The mean hospital stay was significantly longer in Group A (3.2 ± 0.87 days) compared to Group B (1.52 ± 0.58 days), indicating a faster recovery with RIRS. Kanchi et al^18^ similarly found no significant difference in hemoglobin drop between Mini-PCNL (0.88 ± 0.44 g/dL) and RIRS (0.99 ± 0.65 g/dL), but their reported hospital stay was 3.30 ± 0.95 days for Mini-PCNL and 3.07 ± 1.91 days for RIRS. In contrast, Mahmood et al^19^ found a statistically significant difference in hospital stay (Mini-PCNL: 1.18 ± 0.944 days, RIRS: 1.01 ± 0.115 days) but reported a higher hemoglobin drop in Mini-PCNL (0.78 ± 0.49 g/dL) than in RIRS (0.3 ± 0.2 g/dL). Mahmoud et al^26^ also reported a significantly longer hospital stay in Mini-PCNL (2.3 ± 1.5 days) than in RIRS (1.5 ± 0.45 days). Lee et al,^27^ on the other hand, found no significant difference in hemoglobin drop between Mini-PCNL (0.69 ± 0.98 g/dL) and RIRS (0.38 ± 0.97 g/dL). Recent studies confirmed a greater hemoglobin drop and transfusion rate in miniPNL versus retrograde stone management.^20^^-^^22^^,^^28^

Regarding postoperative stent fixation, all patients in both groups had a ureteric catheter or DJ stent. In Group I, 84% of patients had a ureteric catheter, while none in Group B received one. Conversely, 16% of patients in Group A had a DJ stent, whereas all patients in Group B had a DJ stent, showing a significant difference between the groups. Ali et al^29^ similarly found that all RIRS cases required DJ stent placement, which was removed 3-8 weeks postoperatively. Supporting this, Kaya et al^25^ reported that DJ stent placement was 100% in Mini-PCNL and 57% in RIRS. A non-uniform pattern of postoperative drainage may result in outcome variability. However, this is not feasible in real practice as it is based on individual surgeon preferences.

The stone-free rate, defined as no residual stones or fragments smaller than 4 mm, was comparable between the groups. In the study, 92% of patients in Group A and 84% in Group B achieved complete stone clearance, with no significant difference between the groups. Mahmood et al^19^ reported similar findings, with 93.3% of patients in the Mini-PCNL group and 89% in the RIRS group achieving stone-free status. Mahmoud et al^26^ also observed high stone-free rates in both Mini-PCNL (95.6%) and RIRS (88.9%) groups, with no significant difference. Also, 2 recent studies reported higher SFR in the miniPNL group versus the RIRS group in fewer treatment sessions.^20^^,^^22^ Higher detection of smaller fragments might have occurred in the case of routine postoperative CT for all patients in this study. However, the methodology was standardized for both study groups, and this was also the rule for many studies which included postoperative X-ray (KUB) and ultrasonography to document residual stone, while CT (KUB) was resorted to confirm residual stone, as reported by a recent meta-analysis.^30^

Postoperative complications occurred in 28% of patients in Group A and 20% in Group B, but the difference was not statistically significant. The most common complications included fever (16% in Group A vs. 12% in Group B), hematuria, and urinary leakage. Dhayal et al^17^ reported similar complication rates, with 31.7% of Mini-PCNL and 17.5% of RIRS patients experiencing postoperative complications. Kanchi et al^18^ also found no significant difference in complications between the 2 groups.

Despite comparable perioperative incidence of complications between study groups in this study, which was also similar to what was stated by recent meta-analyses.^21^^,^^22^ The authors think that mini-PNL had obviated the risk of devastating ureteral injury associated with RIRS.^21^ Also, supine mini-PNL resulted in a lower intrapelvic renal pressure due to gravity drainage and the presence of the ureter as a side drainage. These advantages of the supine mini-PNL combined with the decreased PNL-related complications with miniaturized PNL ultimately lead to a rise in mini-PNL utility even in stones less than 2 cm in a smaller number of procedures.

Supine miniperc remains very useful and cost-effective in hospitals where RIRS is still limited or cost-restrictive, with fewer consumables, larger size, and more durable LASER fibers. Also, the possibility of pneumatic lithotripsy use and decreased surgeon and anesthesiologist salaries, along with fewer repeat procedures in supine miniPNL in developing countries,^21^ lead to lower miniPNL costs in comparison to RIRS, which is the opposite in developed countries.^20^^,^^22^

### Study Limitations and Future Recommendations

Despite its strengths, the study has several limitations. The sample size was relatively small, which may have affected the statistical power of some findings. Additionally, as a single-center study, the results may not be generalizable to broader populations. The study also lacked long-term follow-up, preventing an assessment of stone recurrence rates and long-term complications. Furthermore, while we focused on stone clearance and operative parameters, we did not evaluate postoperative renal function, pain levels, or patient-reported quality of life. Moreover, although both procedures were compared in terms of clinical and surgical outcomes, cost-effectiveness was not evaluated. This remains a critical consideration in clinical decision-making, especially in resource-limited settings, where economic factors may significantly influence the choice of surgical modality for managing renal stones of this size. Finally, the study did not compare these procedures with other treatment modalities for renal stones, which could have provided a more comprehensive analysis.

Both supine mini-PCNL and RIRS are effective treatment modalities for managing renal stones, with comparable stone-free rates and complications. Mini-PCNL demonstrated a significantly shorter operative time but required a longer hospital stay and higher fluoroscopic exposure. However, bypassing the ureter and going directly into the renal system avoided many ureteral injury-related complications. In contrast, RIRS was associated with a lower fluoroscopic time and shorter hospitalization but had a slightly higher rate of residual stones requiring auxiliary treatment with a higher irrigation pressure. Thus, supine mini-PCNL is still a good competitor of RIRS in the management of stones less than 2 cm.

Future research should include multicenter studies with larger sample sizes and longer follow-up periods to assess the long-term outcomes of Mini-PCNL and RIRS. Incorporating patient-reported outcomes and evaluating renal function postoperatively would provide valuable insights into the overall impact of these procedures.

## Figures and Tables

**Table 1.** Comparison of Baseline Data, Stone Size and HU and Operative Data Between Studied Groups

		**Group A (n = 25)**	**Group B (n = 25)**	***P***
Age (years)		40.8 ± 14.8	40.1 ± 14.6	.85
Sex	Male	17(68%)	18(72%)	.75
	Female	8(32%)	7(28%)	
Side	Right	13 (52%)	14 (56%)	.77
	Left	12 (48%)	11 (44%)	
BMI (kg/m^2^)		23.03 ± 2.7	22.7 ± 2.7	.75
Stone size		15.3 ± 2.2	16 ± 2.3	.70
HU		1317.5 ± 263	1345.8 ± 261.4	.30
Operative data	Operative time (min)	80.6 ± 11.8	93.6 ± 11.6	< .001*
	Flouroscopic time (sec)	334.6 ± 55.7	68.8 ± 13.5	< .001*

Data are presented as mean ± SD or frequency (%).

Group I, mini PCNL; Group II, RIRS group; HU, housefield unit; BMI, body mass index.

*Significant *P* value < .05.

**Table 2.** Comparison of Post-Operative Data Between the Studied Groups

		**Group A (n = 25)**	**Group B (n = 25)**	***P***
Hemoglobin drop (gm/dL)		0.58 ± 0.18	0.68 ± 0.27	.12
Hospital stay		3.2 ± 0.87	1.52 ± 0.58	< .001*
Type of Post-operative	DJ	4 (16.0%)	25 (100%)	< .001*
Stent	Ureteral	21 (84.0%)	0 (0.0%)	

Data are presented as mean ± SD or frequency (%).

Group I, mini PCNL; Group II, RIRS group; DJ, double-J.

*Significant *P* value < .05.

**Table 3.** Comparison of Stone Free Rate and Auxiliary Treatment Between the Studied Groups

		**Group A (n = 25)**	**Group B (n = 25)**	***P***
Stone free rate	Free	23(92.0%)	21(84.0%)	.5
	Residual stone	2(8.0%)	4(16.0%)	
Auxiliary treatment		2(4.0%)	4(16.0%)	.38

Data are presented as frequency (%). Group I, mini PCNL; Group II, RIRS group.

**Table 4.** Comparison of Postoperative Complications Between Studied Groups

		**Group A (n = 25)**	**Group B (n = 25)**	***P***
Complication	Fever	4 (16.0)	3 (12.0)	.23
	Blood transfusion	1 (4.0)	0 (0)	
	Mild sepsis*	0 (0.0)	2 (8.0)	
	Urine leak >12 h	2 (8.0)	0 (0.0)	
Complication grade	Grade I	6 (24.0)	4 (16.0)	.7
	Grade II	1 (4.0)	1 (4.0)	1

Data are presented as frequency (%).

Group I, mini PCNL; Group II, RIRS group; UTI, urinary tract infection.

*Mild sepsis = febrile UTI with no hypotension, shock, or organ dysfunction (QSOFA score < 2).

## Data Availability

The data that support the findings of this study are available on request from the corresponding author.
